# Larval Ascariasis Triggers Unresolved Anemia, Persistent Inflammation, and Chronic Pulmonary Disease after Single and Reinfection in a Dose-Dependent Manner in Mice

**DOI:** 10.1021/acsinfecdis.5c00477

**Published:** 2025-09-12

**Authors:** Jorge Lucas Nascimento Souza, Chiara Cássia Oliveira Amorim, Camila de Almeida Lopes, Flaviane Vieira-Santos, Ana Rafaela Antunes-Porto, Fernanda Rezende Souza, Evelyn Ane Oliveira, Thaynan Cunha Vieira, Lucas Kraemer, Marcelo Eduardo Cardozo, Ramayana Morais de Medeiros Brito, Luisa Mourão Dias Magalhães, Geovanni Dantas Cassali, Ricardo Toshio Fujiwara, Remo Castro Russo, Lilian Lacerda Bueno

**Affiliations:** † Laboratory of Immunobiology and Control of Parasites, Department of Parasitology, Institute of Biological Sciences, 28114 Universidade Federal de Minas Gerais, Belo Horizonte, Minas Gerais 31270-901, Brazil; ‡ Laboratory of Comparative Pathology, Department of Pathology, Institute of Biological Sciences, Universidade Federal de Minas Gerais, Belo Horizonte, Minas Gerais 31270-901, Brazil; § Laboratory of Interactions in Immunoparasitology, Department of Parasitology, Institute of Biological Sciences, Universidade Federal de Minas Gerais, Belo Horizonte, Minas Gerais 31270-901, Brazil; ∥ Laboratory of Pulmonary Immunology and Mechanics, Department of Physiology and Biophysics, Institute of Biological Sciences, Universidade Federal de Minas Gerais, Belo Horizonte, Minas Gerais 31270-901, Brazil

**Keywords:** chronic inflammation, larval ascariasis, lung disease, murine model, comorbidities, ACO

## Abstract

Although different studies have investigated the pathophysiological aspects of ascariasis using experimental models, the long-term effects following the peak of larval migration in the lungs remain poorly understood, especially in different doses of infection, such as those that mimic better the natural infection scenario. In this study, we evaluated the impact of different infection doses (250, 1250, and 2500 eggs) and amount of exposure (single vs reinfection) on the host’s immune response over an extended period. Our findings demonstrate that even the lowest dose (250 eggs) can induce persistent, though less severe, pathological damages compared to higher doses. These include anemia resulting from alveolar hemorrhage and the development of chronic pulmonary disease. Notably, while lower doses elicit a milder immune response, clinical manifestations tend to appear later, indicating a delayed pathological impact. Importantly, inflammatory infiltrates and altered cytokine levels (including Th1/Th2/Th17) were still observed in the lungs more than 100 days postinfection, even after larval clearance. The humoral immune response against *Ascaris suum* remained active for at least 100 days postinfection, with the potential for longer persistence. Notably, even with lower doses, only two exposures were sufficient to trigger an immune pattern similar to that observed at higher doses. These findings highlight the need for further investigation into the chronic inflammatory effects of *Ascaris* infection, including its impact on distant organs and its potential contribution to the development of noncommunicable diseases and comorbidities. A better understanding of these mechanisms is essential for developing improved strategies to control ascariasis in endemic areas.

## Graphical abstract

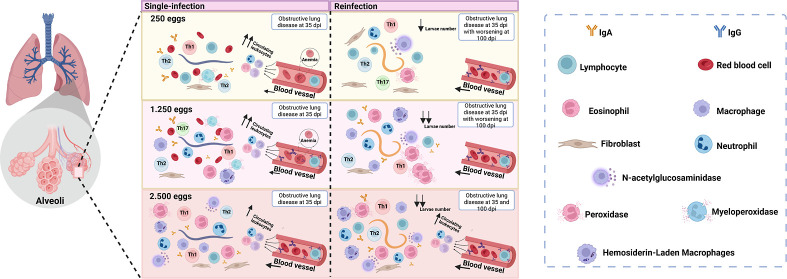

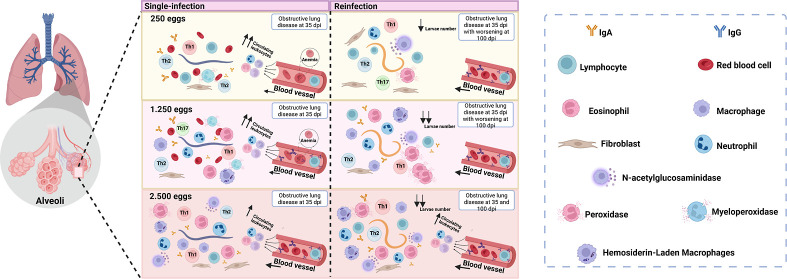

Ascariasis is a neglected tropical disease and the most common helminth infection worldwide.^1^ It is caused by *Ascaris lumbricoides* (Linnaeus, 1758) or *Ascaris suum* (Goeze, 1782).^2–4^ The disease is associated with low socioeconomic and precarious health conditions, mainly in tropical and subtropical regions of developing countries, particularly in Central and Southeast Asia, Latin America, and sub-Saharan Africa.^1,5^ Ascariasis is found in more than 150 countries, with approximately 800 million people infected and causing the loss of nearly 800 thousand years of disability-adjusted life years.^6–8^

Infection with *Ascaris* spp. occurs through the oral ingestion of eggs containing L3 larvae present in the environment. Upon egg ingestion, *Ascaris* spp. larvae hatch in the intestine and start the migration of parasitic larvae stages through tissues (larval ascariasis or acute ascariasis), such as intestinal mucosa, blood circulation, liver, and lungs, and then swallowed again, reaching the small intestine where the parasite maturates into an adult worm in the lumen.^9,10^ In general, ascariasis triggers an intense local and systemic inflammatory response during the migration of larvae through tissues.^2,10^ Many studies have characterized the response induced by ascariasis as Th2-dependent with the presence of regulatory cytokines.^11–13^ However, some studies in animal models have shown different inflammatory responses depending on the amount of parasite exposure, with the expression of TNF and IL-6 cytokines in a single-infection^2^ or a mixed Th2/Th17 immune response predominating in multiple infections.^10^

Although the inflammatory response triggered by ascariasis induces protection and promotes control of parasite burden, it can also damage the host organs. A recent review demonstrated that increasing data related to ascariasis discuss the consequences beyond infection, including various clinical conditions, which points toward numerous unknown and complex factors in ascariasis research.^7^ This included a relationship with pulmonary allergic inflammation,^14,15^ pneumonia due to intense eosinophilia,^16^ asthma-like conditions,^17,18^ pulmonary fibrosis,^10,19,20^ and recently, lung manifestation similar to chronic obstructive pulmonary disease (COPD) due to emphysema in senescent infected mice.^21^

On the other hand, most of these studies in the experimental model explore the immune physiopathological aspects at the peak of larval migration in tissues,^2,10^ few have explored later times after the migration, which demonstrate the persistence of an inflammatory response even in the absence of larvae,^21,22^ and all these evaluate the infection using high standard doses of 2500 eggs, while only a few evaluated the infection with lower doses such 100, 500, and 1000 ^23^ or 25 and 250.^24^ In this sense, significant gaps remain related in the immunopathological aspects of *A. suum* experimental infection, particularly regarding the long-term consequences after the peak of larval migration in the lungs and the impact of different infection doses that better reflect real-world scenarios. Notably, lower infection doses have been shown to elicit immune responses that more closely mimic natural infections.^24^ Therefore, in this study, we characterize the physiopathological and immunological aspects of experimental infection by *A. suum* in a murine model, focusing on infection dose (250, 1250, and 2500 eggs) and exposure frequency (single infection and reinfection) after the peak of larval migration in the lung and describe the damage caused to the host in a context of long-term chronic inflammation.

## 2 Results

### 2.1 Impact of Infection Dose and Exposure Frequency on Body Weight and Clinical Manifestation

Body weight and clinical changes (piloerection, tremors, abnormal breathing, and trunk flexion) were monitored up to the 100th day postinfection (dpi) to assess the impact of different doses of infection and the number of exposures in the host. However, data were presented only up to day 80 (Figure 1B,C) to highlight the period in which body weight stabilized across all groups, as later time points mainly reflect continued weight gain after stabilization. Mice infected with the highest dose (2500 eggs) showed an abrupt decline in weight from the second until the 10th dpi (from 16th to 24th follow up day), corresponding to the larval migration period. On the other hand, mice exposed twice (RE group) to 2500 eggs also showed weight loss after the second exposure (14th dpi) but regained it more rapidly than other groups (Figure 1B). Mice infected with the intermediate dose (1250 eggs), regardless of the number of exposures, lost weight during the larval migration period and experienced moderate weight loss during the same period. No weight loss was observed in the groups receiving the lowest dose (250 eggs). Groups infected with 250 and 1250 eggs showed delayed weight gain, reaching control levels only by 46 dpi in the 1250-dose group (60th follow-up day), while in the 250-dose group, weight gain aligned with the control by 50 dpi, or 62nd follow-up day (SI 250) and 54 dpi (RE 250) (Figure 1B). The RE 1250 group specifically lost weight from 6 to 12 dpi and resumed gain by 14 dpi. Clinical alterations were observed in the 2500 and 1250 groups starting at 8 dpi, lasting until 10 dpi (SI 1250) or 12 dpi (SI/RE 2500 and RE 1250) (Figure 1C). All evaluated clinical alterations were present only in 2500 groups, while the 1250 groups showed piloerection and abnormal breathing. After re-exposure, only the RE 2500 group showed clinical signs (limited to piloerection) from 6 to 10 dpi (Figure 1C). Animals in the 250-dose group showed no clinical alterations, presenting a score of 0 throughout the experiment (Figure 1C).

**Figure 1:**
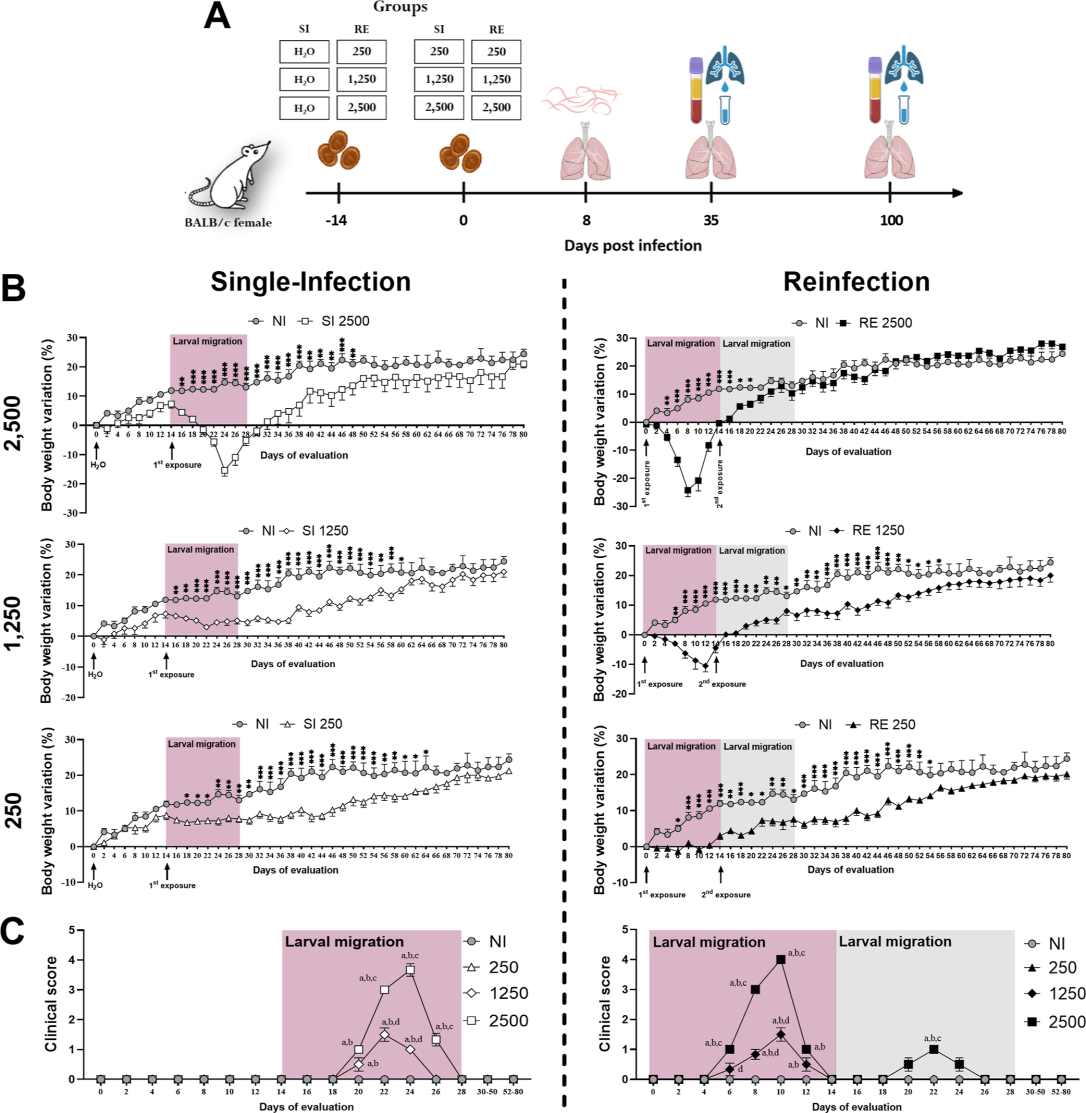
Experimental design and mice alterations postinfection by *A. suum* according to the dose of infection and the amount of exposure. (A) Experimental design of the *A. suum* infection in mice according to different infection doses and amount of exposures and euthanasia at 35 dpi (*n* = 8, per group) and 100 dpi (*n* = 8 per group). (B) Body weight variation throughout 80 days of evaluation according to different doses and amount of exposure. (C) Evaluation of clinical alterations throughout 80 days. Highlights in red indicate the period of larval migration in the host after the first exposure, while gray highlights indicate the period of larval migration in the host after the second exposure. Arrows indicate the exact moment of gavage (filtered water or infection) according to the evaluation day. In B, results are expressed in percentages from the initial weight of the mice before the experimental design, and multiple *t* tests were used to evaluate variations in body weight. In C, the two-way ANOVA test, followed by the multiple comparisons test, was used to assess variations in clinical signs. The results are shown as mean ± SEM, and statistical differences are represented by an asterisk, where * represents differences between groups, where *p* < 0.05, **p < 0.01, and ***p < 0.001 or and (a) represent significant differences (*p* ≤ 0.05) with the NI group, (b) 250 group, (c) 1250 group, and (d) 2500 group.

### 2.2 Lowest Parasite Burden of *A. suum* Results in Anemia in Single-Infected Mice due to Persistent Alveolar Hemorrhage

After observing that infection dynamics varies with dose and exposure, we quantified the larvae that reached the lungs at different doses and after reinfection. To this end, mice were euthanized to collect the lungs and bronchoalveolar lavage (BAL) for larval counting at 8 dpi, corresponding to the peak of larval migration.^2^ In the SI group, mice infected with 2500 eggs had a mean of 200 larvae in the lungs, while infections with 1250 and 250 eggs resulted in lower counts (100 and 5 larvae, respectively) (Figure 2A). Upon reinfection, larval burden significantly dropped across all doses, with reductions of 96.94%, 96.97%, and 85.71% for the 2500, 1250, and 250 groups, respectively.

**Figure 2:**
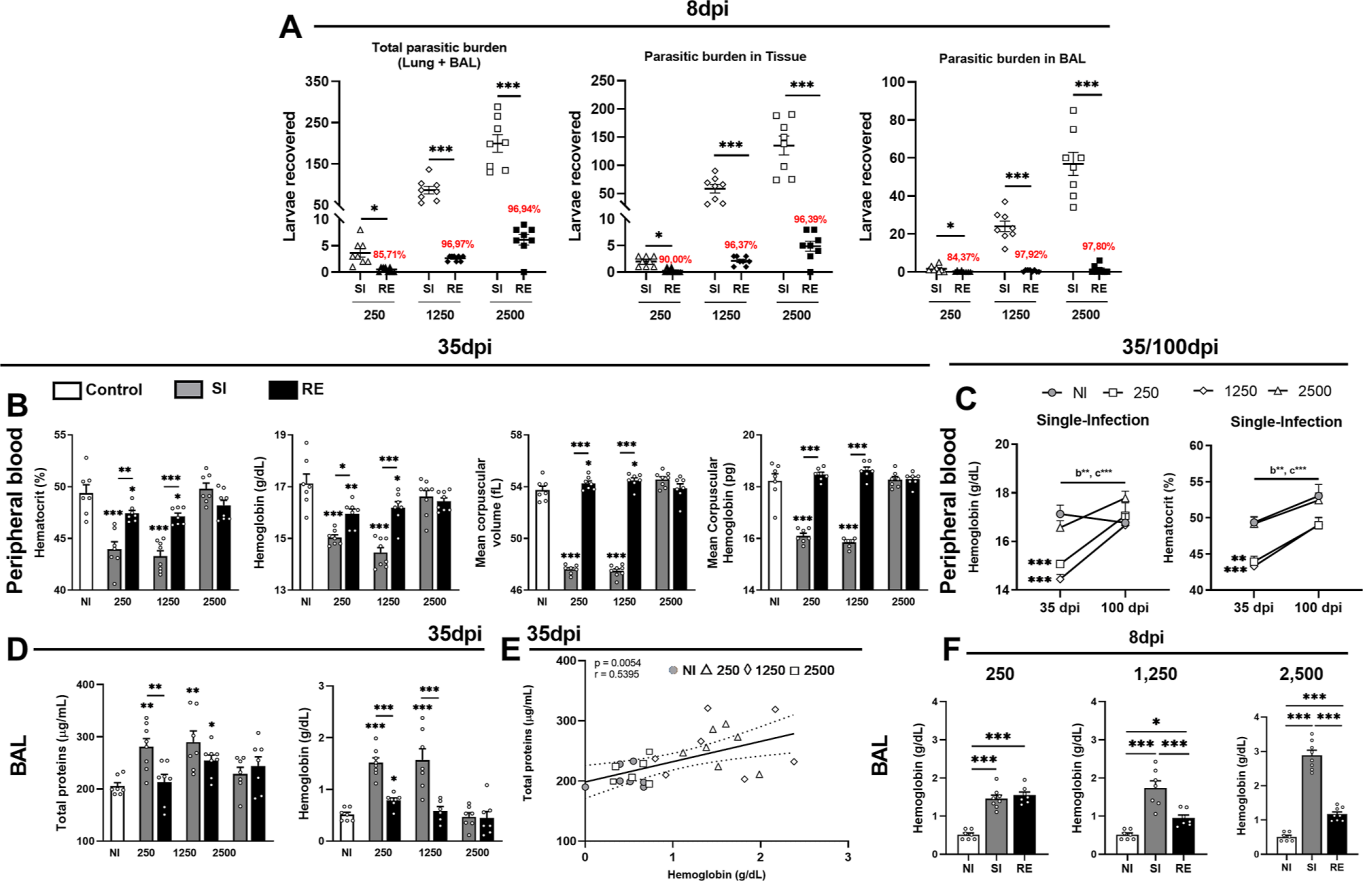
Larvae recovered from lung and BAL, hematological analysis, and characterization of alveolar hemorrhage among groups of mice BALB/c infected by *A. suum* in different doses and amount of exposures at 35 dpi and 100 dpi. (A) Number of larvae recovered from the lung and BAL. (B) Hematological changes including hemoglobin dosage, hematocrit determination, mean corpuscular volume calculation, and mean corpuscular hemoglobin calculation according to different doses and amount of exposure at 35 dpi. (C) Variation of hemoglobin and hematocrit in single-infection groups at 35 dpi and 100 dpi. (D) Levels of total proteins and hemoglobin in BAL at 35 dpi. (E) Correlation between levels to total protein and hemoglobin in BAL in groups single-infected. (F) Levels of hemoglobin in BAL at 8 dpi per dose. Results are expressed in mean ± SEM. Red values in A indicate a reduction (%) in larvae compared to the first exposure, and a *t*-test was used to evaluate differences between groups (*n* = 8 per group). In B, C, D, and F, the one-way ANOVA test, followed by Tukey’s test, was used to evaluate differences among NI, SI, and RE groups in each dose or between times (*n* = 8 per group). In E, the Pearson correlation test was used to verify the correlation between the levels of total protein and hemoglobin in BAL. Statistical differences are represented by an asterisk, where * without the bar represents differences with the NI group and differences between the amount of exposure (SI × RE) in respective dose are represented by the asterisk with the bar where *p* < 0.05, **p < 0.01, and ***p < 0.001 or in C, the bar indicates the differences between the groups in 35 and 100 dpi where (a) represents significant differences of NI group, (b) 250 group, (c) 1250 group, and (d) 2500 group.

At 35 dpi, mice from the SI 250 and 1250 groups presented microcytic, hypochromic anemia, confirmed by reduced hemoglobin, hematocrit, MCV, and MCH (Figure 2B). These alterations were absent in SI 2500 and all RE groups (Figure 2B). By 100 dpi, anemia had resolved in SI 250 and 1250 groups, coinciding with weight recovery (Figures 1B and 2C). Hematological values in SI 2500 remained comparable to the NI group.

Given the delayed recovery and anemia in SI 250 and 1250 groups, we hypothesized ongoing alveolar hemorrhage at 35 dpi. Supporting this, BAL fluid showed elevated total protein and hemoglobin levels in these groups, positively correlated with systemic hemoglobin and protein concentrations (Figure 2D–E). To complement our analyses and better understand the reduced hemoglobin levels observed in animals infected with the highest dose, we measured hemoglobin levels in the BAL at 8 dpi (Figure 2F). Alveolar hemorrhage was present at the peak of infection across all doses, with greater intensity in the 2500 egg group (Figure 2F). However, by 35 dpi, hemoglobin levels in the BAL had decreased in the high-dose group, while remaining elevated in mice infected with the lower doses (1250 and 250 eggs), indicating prolonged alveolar damage in these groups.

### 2.3 Infection by *A. suum*, Regardless of Infectious Dose, Leads to an Increase in Leukocytes in the BAL That Persists for up to 100 days

Leukocyte counts in peripheral blood revealed increased levels only in SI groups and RE 2500 at 35 dpi (Figure 3A). Eosinophils levels increase in all infected groups, with differences only between SI/RE groups in the 250 groups, while neutrophils increased only in the SI 250/1250 groups, monocytes increased in RE 250 and SI 1250 groups, and lymphocytes increased only in the SI 1250 group (Figure 3A). The leukocyte count after 100 dpi was similar to that of the control group in both SI/RE groups (data not shown). In addition, platelets of SI 250/1250 mice showed a substantial increase compared to the control group and reinfected group that received the same doses (Figure 3B).

**Figure 3:**
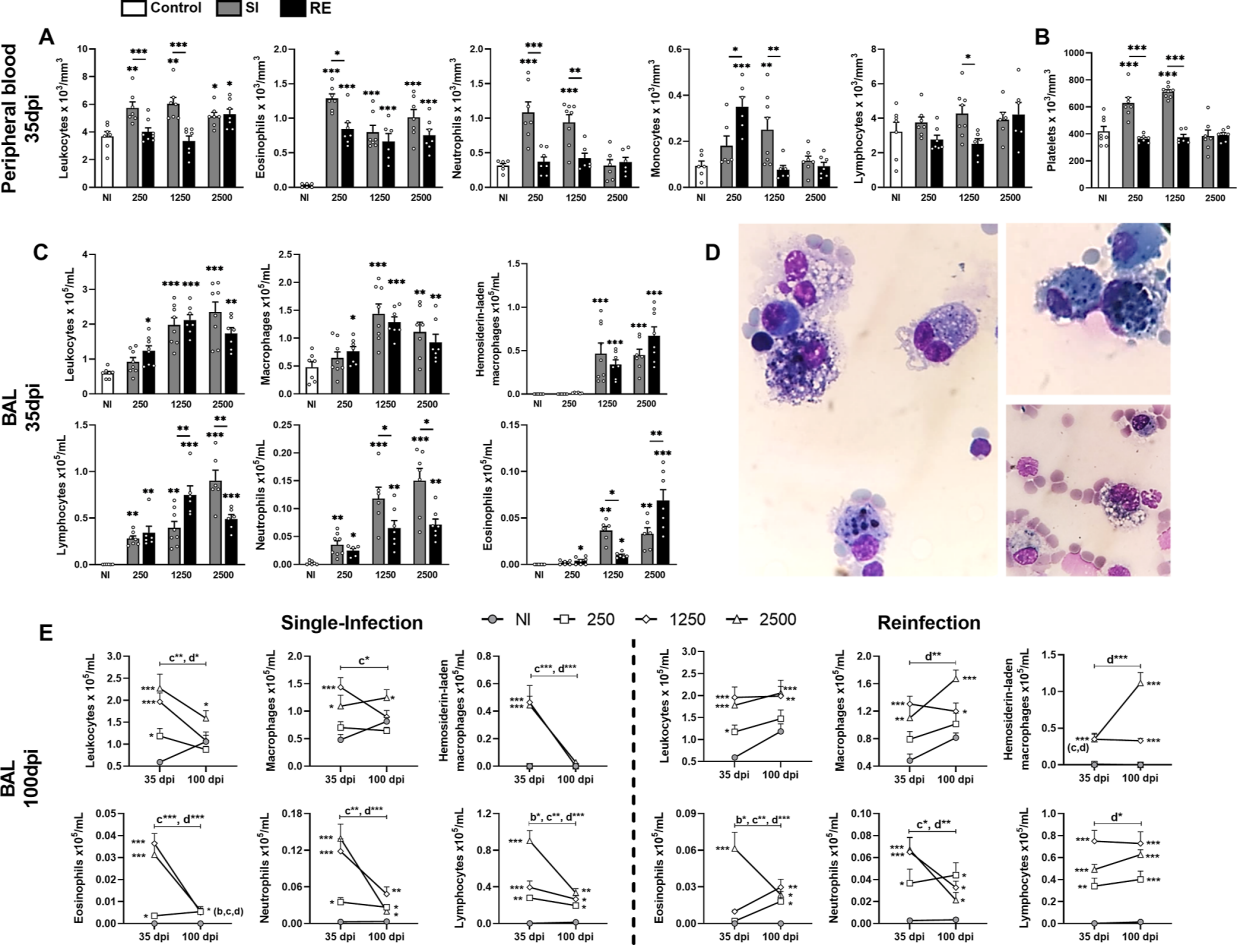
Blood leukocyte counts, platelets, and evaluation of leukocyte counts in BAL from mice infected with *A. suum* at different days postinfection according to different doses or amounts of exposures. (A) Blood leukocyte counts including eosinophils, neutrophils, monocytes, and lymphocytes count at 35 dpi. (B) Platelets counts. (C) Leukocyte counts in BAL including macrophages, hemosiderin-laden macrophages, lymphocytes, neutrophils, and eosinophils count at 35 dpi. (D) Hemosiderin-laden alveolar macrophages observed in mice infected with 1250 and 2500 larvated eggs of *A. suum*. (E) Leukocyte counts in BAL including, macrophages, hemosiderin-laden macrophages, lymphocytes, neutrophils, and eosinophils count at 100 dpi divided according to the amount of exposure. A one-way ANOVA test followed by Tukey’s test was used to evaluate differences among NI, SI, and RE groups in each dose (*n* = 8 per group). The results are shown as the mean ± SEM, and statistical differences are represented by an asterisk, where * without the bar represents differences between the NI group and differences between the amount of exposure (SI × RE) in respective doses are represented by the asterisk with the bar and *p* < 0.05, **p < 0.01, and ***p < 0.001 or in E, the bar indicates differences between the groups in 35 and 100 dpi where (a) represents significant differences of the NI group, (b) 250 group, (c) 1250 group, and (d) 2500 group.

Leukocytes levels in BAL were assessed at 35 and 100 dpi. Total cell counts increased in the groups infected with 1250/2500 eggs, both SI/RE groups (Figure 3C). Macrophages increased in these groups with atypical morphology (hemosiderin-laden macrophages), likely due to hemorrhage. These cells were more prevalent in the 1250/2500 groups (Figure 3C,D). Lymphocytes increased in all groups, especially in SI/RE 250, RE 1250, and SI 2500, while neutrophils were significantly increased in SI 250/2 groups, and eosinophils were elevated in RE 2500 and SI 1250 (Figure 3C). At 100 dpi, leukocyte levels normalized in most SI groups, except SI 2500 and RE 1250/2500, which remained elevated. Hemosiderin-laden macrophages persisted in these groups and increased in RE 2500 (Figure 3E).

### 2.4 Higher Doses of Eggs Favor the Persistent Cell Recruitment and Expansion of the Inflammatory Infiltrate in the Lung, Which Seems to be Related to Rapid Tissue Repair

Given that higher doses and more exposures may enhance tissue repair through increased cell recruitment, we assessed lung inflammation in the mice. As expected, *A. suum* infection triggered inflammation that was still presented at 35 dpi in all groups, but the intensity varied (Figure 4). NAG and EPO levels increased gradually with higher egg doses and more exposures, especially in the SI and RE groups with highest doses and the RE 1250 group. In contrast, mice in SI groups with lower doses did not show increased NAG and EPO activity (Figure 4A). Additionally, MPO activity increased in the SI 250/1250 groups, while the other groups had similar levels to the control group (Figure 4A).

**Figure 4:**
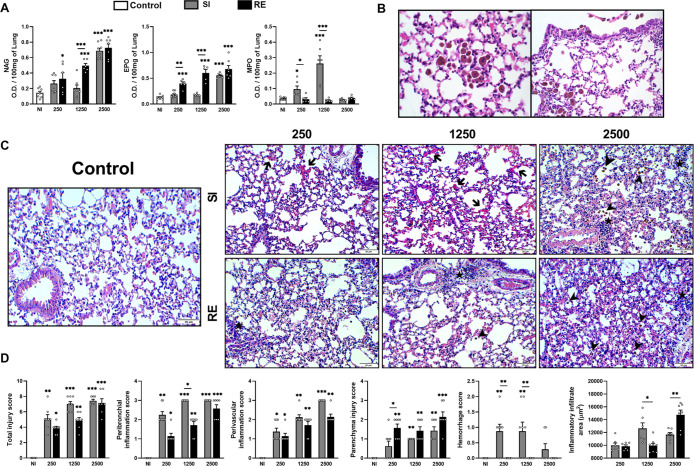
Characterization of the cellularity of lung tissue of mice infected with *A. suum* at 35 dpi. (A) Indirect activity of macrophages (NAG), eosinophils (EPO), and neutrophils (MPO) in lung tissue. (B) Hemosiderin-laden alveolar macrophages observed in lung tissue at 35 dpi. (C) Representative hematoxylin and eosin staining of lung sections where hemorrhage area (arrows), hemosiderin-laden alveolar macrophages (arrowhead), inflammatory infiltrate (★), and bar = 30 μm. (D) Scores of lung inflammation in lung tissue and evaluation of inflammatory infiltrate areas. One-way ANOVA test followed by Tukey’s test was used in A and D Kruskal–Wallis test followed by Dunn’s test were used to evaluate differences among NI, SI, and RE groups in each dose (*n* = 8 per group). In the evaluation of inflammatory infiltrate areas in D, a *t*-test was used between the amount of exposure (SI × RE) in the respective dose of infection. The results are shown as the mean ± SEM, and statistical differences are represented by an asterisk, where * without the bar represents differences between the NI group and differences between the amount of exposure (SI × RE) in respective doses are represented by the asterisk with the bar and *p* < 0.05, **p < 0.01, and ***p < 0.001.

The histopathological scores were similar to those observed in BAL. Hemosiderin-laden macrophages were observed in tissues from the 250/2500 egg groups (Figure 4B,C). At 35 dpi, all infected groups displayed inflammatory infiltration, vascular integrity loss, and edema, with more extensive inflammation areas in the 250/2500 egg groups (Figure 4D). Peribronchial and perivascular inflammation was more intense in SI groups than RE groups, while parenchymal inflammation was greater in RE groups, and hemorrhage occurred mainly in the SI 250/1250 groups (Figure 4D). We found that inflammation areas were larger in the SI 1250 and RE 2500 groups. All groups showed inflammatory infiltrates with polymorphonuclear and mononuclear cells around blood vessels and airways, along with reduced thickening of alveolar septa. Macrophage presence was stronger in the 250/2500 groups and increased with exposure. While no clear difference was found between SI/RE groups in histopathological scores, morphometric analysis supported the NAG and EPO results (Figure 4A,D). Overall, *A. suum* infection led to chronic lesions by 35 dpi, with the intensity varying by dose and exposure frequency. At 100 dpi, lung tissue analysis revealed inflammation but with less intensity than at 35 dpi (Figure S1). NAG, EPO, and MPO levels showed no significant differences from the control group, and their activity decreased compared to 35 dpi (data not shown).

At 35 dpi, we analyzed the formation of lung fibrosis to understand the repair dynamics. In the SI groups, fibrosis areas increased with higher infectious doses (Figure 5A–D). However, in RE groups, lower doses showed more evident fibrosis compared to groups that received 250/2500 eggs (Figure 5E–G). Morphometric analysis confirmed that RE 250 animals had more fibrosis than SI 250 animals. For the 1250 egg dose, exposure frequency did not affect fibrosis, but in the 2500 group, fibrosis was more intense in SI compared to RE groups (Figure 5H). Together, these findings suggest persistent immune activation and tissue adaptation, influenced by dose and exposure.

**Figure 5:**
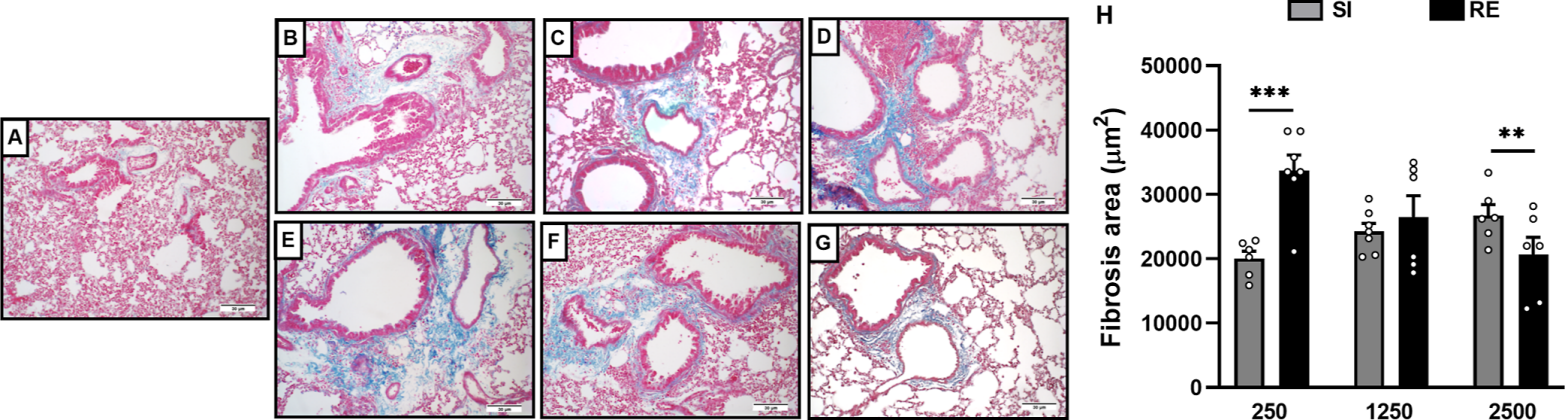
Evaluation of fibrosis area in the lung of mice infected by *A. suum* at 35 dpi. (A) Representative Masson’s trichrome staining in the lung of the NI group. (B) Representative Masson’s trichrome staining in the lung of the SI 250 group. (C) Representative Masson’s trichrome staining in lung of the SI 1250 group. (D) Representative Masson’s trichrome staining in the lung of the SI 2500 group. (E) Representative Masson’s trichrome staining in the lung of the RE 250 group. (F) Representative Masson’s trichrome staining in the lung of the RE 1250 group. (G) Representative Masson’s trichrome staining in the lung of the RE 1250 group. (H) Analysis of the area of fibrosis formation. *t*-Test was used to evaluate differences between groups between the amount of exposure (SI × RE) in respective doses (*n* = 8 per group). The results are shown as the mean ± SEM, and statistical differences are represented by an asterisk, where * with the bar indicates differences between the groups in respective dose, where *p* < 0.05, **p < 0.01, and ***p < 0.001.

### 2.5 Lowest Doses of Infection Promote a Significant Increase in Regulatory Cytokines in the Lung and Influence the Production of Mixed Th1/Th2/Th17 Cytokines, While Higher Doses Lead to Persistently Elevated Cytokine Levels at 100 dpi

Based on the inflammatory infiltrates, we analyzed cytokine profiles in the lungs at 35 and 100 dpi (Figure 6). The results showed significant increases in TNF, IL-5, and IL-10 in SI 250 and 1250 groups, with IL-5 and IL-10 being highest in SI 250 and TNF in SI 1250 (Figure 6B,C,G). IL-13 increased only in SI 250 (Figure 6E), while IL-17A was elevated in RE 250 and SI 1250 groups (Figure 6F). TGF-β increased in all 250-dose groups and in SI 1250 and RE 2500 (Figure 6H). IL-6 was high across all groups (Figure 6D), and IL-2 levels decreased significantly in SI 2500 and RE 2500 (Figure 6A). IFN-γ and IL-4 were undetectable at 35 dpi. These findings highlight the mixed immune response to *A. suum*, with lower doses maintaining high levels of type 1 and 2 cytokines, while the 2500-dose groups had a faster immune response due to higher antigen levels.

**Figure 6:**
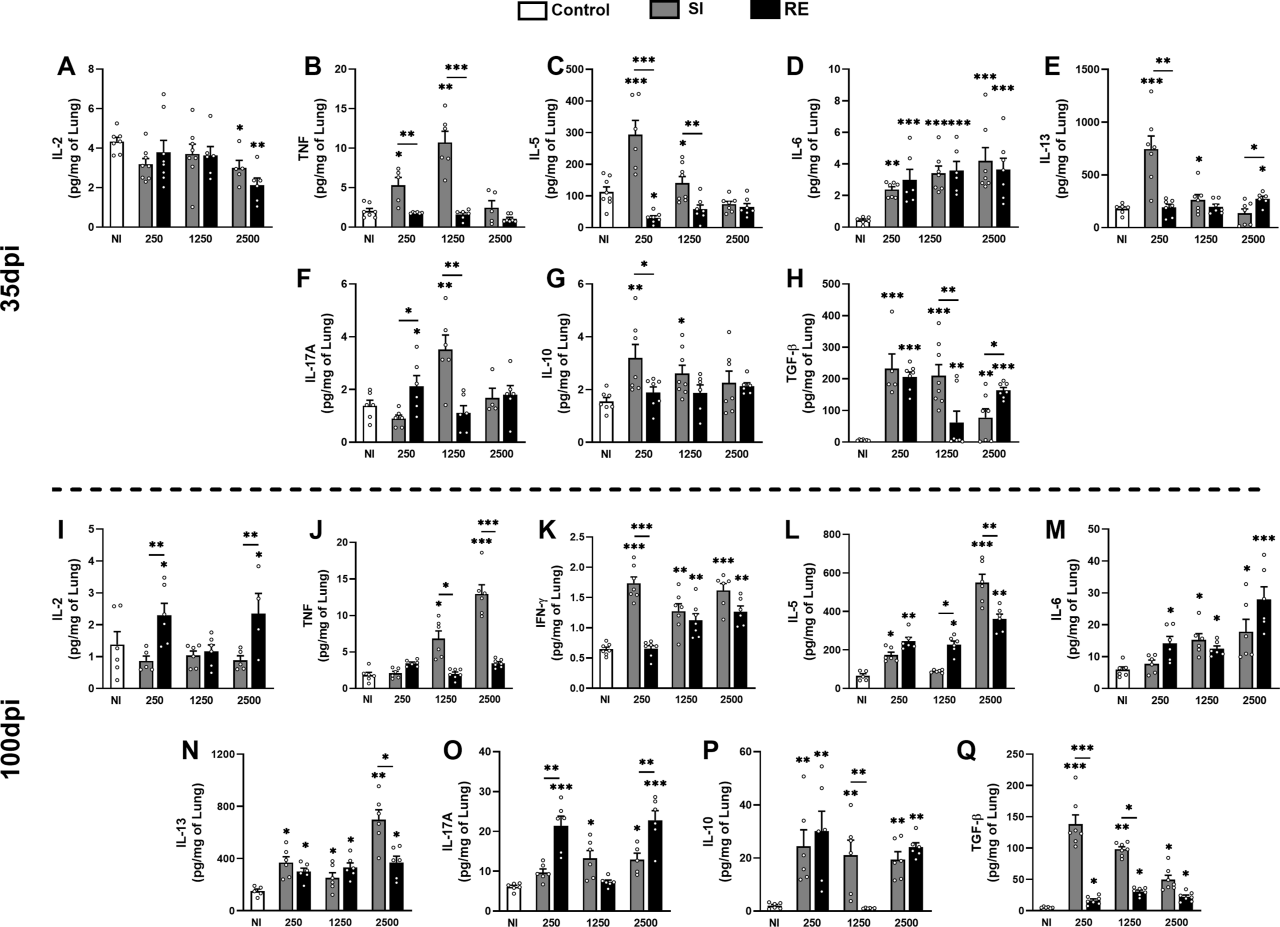
Profile of cytokines present in lung tissue of mice BALB/c infected with *A. suum* at 35 and 100 dpi according to the different doses and amount of exposure. (A) Levels of IL-2 at 35 dpi; (B) TNF-α at 35 dpi; (C) IL-5 at 35 dpi; (D) IL-6 at 35 dpi; (E) IL-13 at 35 dpi; (F) IL-17A at 35 dpi; (G) IL-10 at 35 dpi; (H) TGF-β at 35 dpi; (I) IL-2 at 100 dpi; (J) TNF-α at 100 dpi; (K) IFN-γ at 100 dpi; (L) IL-5 at 100 dpi; (M) IL[1]­6 at 100 dpi; (N) IL-13 at 100 dpi; (O) IL-17A at 100 dpi; (P) IL-10 at 100 dpi; and (Q) TGF-β at 100 dpi. The results are shown as the mean ± SEM. A one-way ANOVA test followed by Tukey’s test was used to evaluate differences among NI, SI, and RE groups in each dose (*n* = 8 per group). Statistical differences are represented by an asterisk, where * without the bar represents differences between the NI group and differences between the amount of exposure (SI × RE) in respective doses are represented by an asterisk with the bar and *p* < 0.05, **p < 0.01, and ***p < 0.001.

At 100 dpi, we observed distinct cytokine patterns across the experimental groups. IL-2 and IL-17A were elevated in the RE 250/2500 groups (Figure 6I,O), indicating sustained immune activation. TNF showed a dose-dependent increase in the SI 1250/2500 groups (Figure 6J), suggesting a continued pro-inflammatory response. IFN-γ was significantly higher in the SI 250 group and in both SI/RE groups that received 1250 or 2500 eggs (Figure 6K). A persistent type 2 response was marked by elevated IL-5 in the SI/RE 250 groups, as well as in RE 1250 and SI/RE 2500 groups (Figure 6L), with IL-13 being most elevated in SI 2500 but high across all groups (Figure 6N). IL-4 remained undetectable across all groups (data not shown). IL-6 was elevated in all groups, with the highest levels in RE 2500 (Figure 6M). Regulatory cytokines such as IL-10 were notably increased in the SI/RE 250 groups and in SI 1250/2500 and RE 2500 groups (Figure 6P), highlighting an ongoing regulatory immune response. TGF-β was elevated in all groups, with the highest levels in the SI groups, showing a dose-dependent decrease from 250 to 2500 eggs (Figure 6Q), where the SI 250 group exhibited the highest levels.

### 2.6 Persistent Inflammation Triggered by *A. suum* Infection Results in Chronic Obstructive Lung Disease

At 35 dpi, spirometry revealed that *A. suum* infection alters lung physiology regardless of dose or exposure number (Figure 7A). All infected groups (except SI 1250) showed reduced pulmonary resistance (Rl), suggesting lung dysfunction. Increases in the tidal volume (TV), inspiratory capacity (IC), and both static and dynamic compliance (Cchord and Cdyn) were observed, indicating altered lung mechanics. Elevated Tiffeneau-Pinelli index (FEV50/forced vital capacity [FVC]) values across groups pointed to an obstructive pattern (Figure 7A). At 100 dpi, different recovery dynamics were observed (Figure 7B). Rl increased in RE 250 and RE 1250 groups and remained elevated in SI 2500, suggesting delayed or impaired resolution. TV was reduced in groups exposed to 2500 eggs, as well as SI 250 and RE 1250 (Figure 7B), reflecting persistent impairment. However, the IC increased in all RE groups, indicating compensatory adaptation (Figure 7B). The Tiffeneau index remained elevated in all RE groups (Figure 7B), reinforcing the presence of an ongoing obstructive dysfunction. While Cdyn improved in RE 250/1250, it decreased in all groups at 100 dpi, with no signs of recovery compared to 35 dpi (Figure 7B). Additionally, RE 2500 showed sustained high Cchord values, suggesting less favorable structural recovery. Together, these findings confirm that *A. suum* infection, especially at higher doses or after reinfection, induces chronic, dose-dependent impairments in lung function that persist over time.

**Figure 7:**
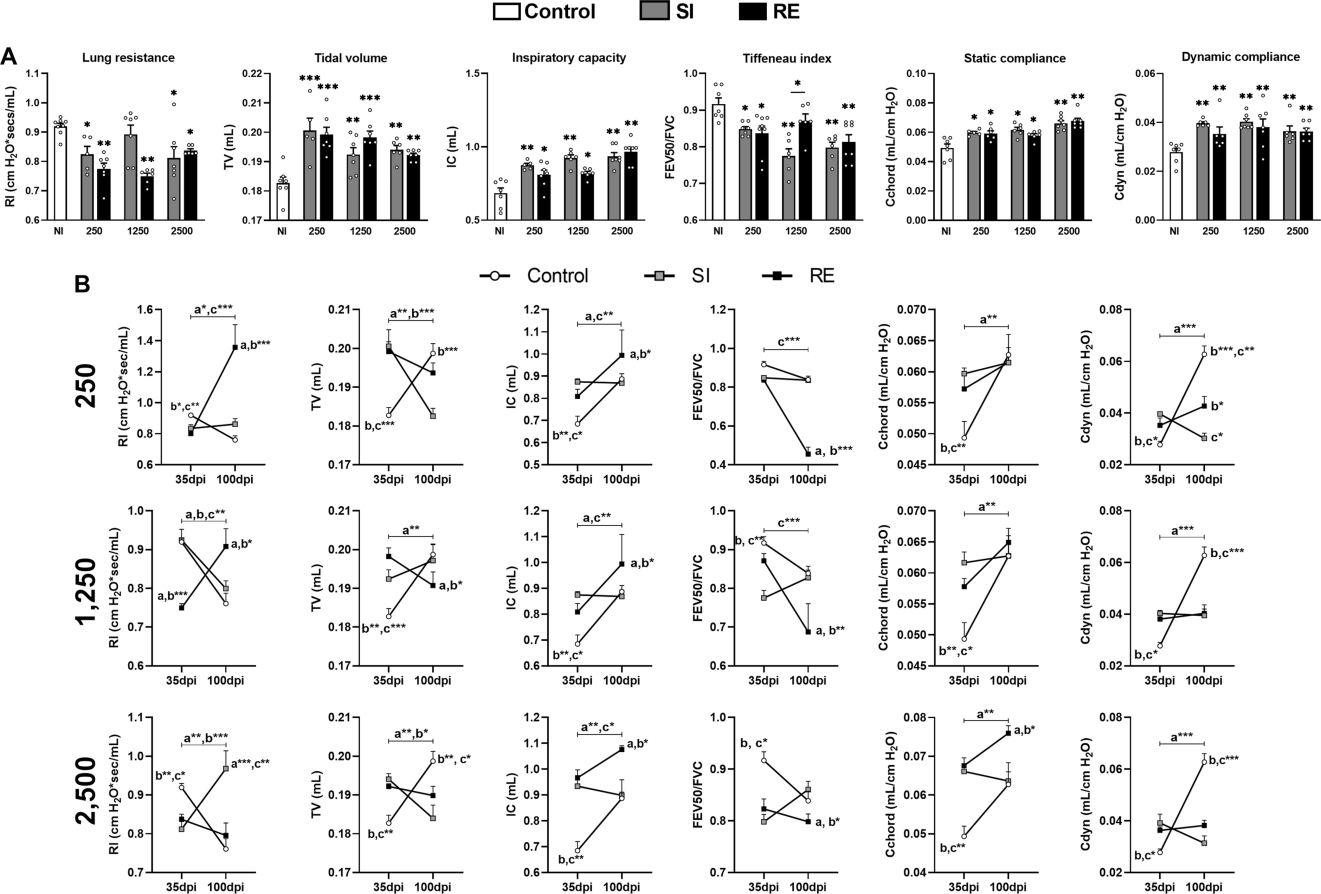
Changes in respiratory mechanics at 35 and 100 dpi according to the amount of exposure and between the same doses. (A) Parameters evaluated in spirometry at 35 dpi including lung resistance, TV, IC, Tiffeneau index, static, and dynamic compliance. (B) Parameters evaluated in spirometry at 35 and 100 dpi divided into infection doses, and parameters such as lung resistance, TV, IC, Tiffeneau index, static, and dynamic compliance. A one-way ANOVA test followed by Tukey’s test was used to evaluate differences among NI, SI, and RE groups in each dose (*n* = 8 per group). The results are shown as the mean ± SEM, and statistical differences are represented by the asterisk, where * without the bar represents differences between the NI group and differences between the amount of exposure (SI × RE) in respective doses are represented by the asterisk with the bar and *p* < 0.05, **p < 0.01, and ***p < 0.001 or in B, the bar indicates differences between the groups in 35 and 100 dpi where (a) represents significant differences of the NI group, (b) 250 group, (c) 1250 group, and (d) 2500 group.

### 2.7 Lowest Doses Promote an Increase in Antibody Levels in Reinfection Similar to the Highest Doses and Persist for 100 dpi

The assessment of antibody levels revealed important insights into the humoral immune response up to 100 dpi. At 35 dpi, infection with 1250 or 2500 eggs led to elevated total anti-*Ascaris* IgG levels, regardless of single or repeated exposure. In contrast, a low dose (250 eggs) induced increased *Ascaris*-specific IgG levels only after re-exposure, highlighting the importance of both dose and antigenic boosting in humoral activation (Figure 8A). For anti-*Ascaris* IgA in BAL, the SI groups displayed similar levels across doses. However, RE groups exhibited a dose-dependent pattern: RE 250 and RE 1250 showed decreased IgA compared to their respective SI groups, while RE 2500 showed higher IgA levels than SI 2500 (Figure 8A).

**Figure 8:**
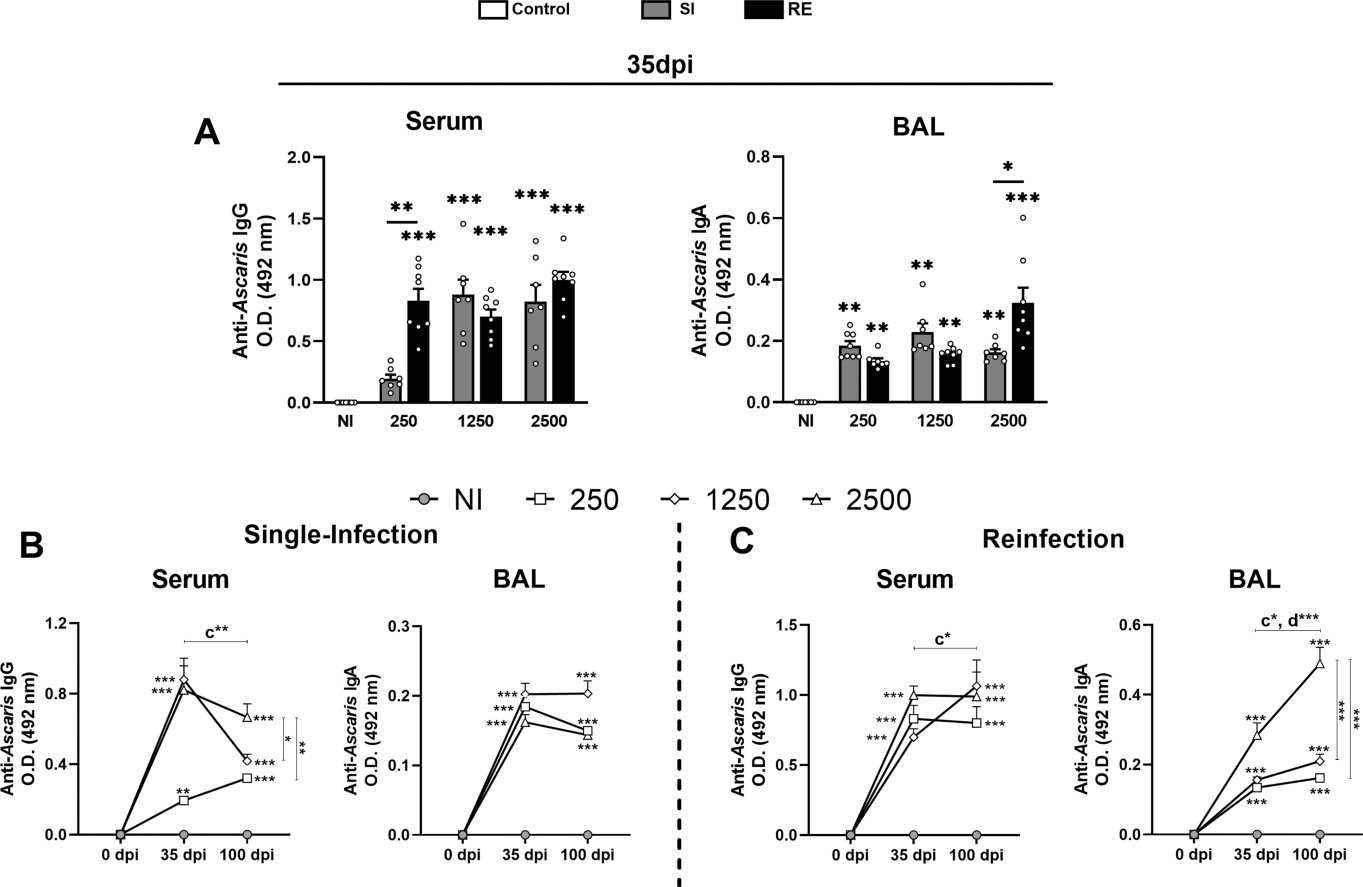
Evaluation of antibody anti-*Ascaris* responses at 35 and 100 dpi according to different doses and amounts of exposure. (A) Determination of the presence of anti-*Ascaris* IgG (O.D 492 nm) in serum and anti-*Ascaris* IgA (O.D 492 nm) in BAL of animals according to different doses in the SI and RE groups at 35 dpi. (B) Determination of the presence of anti-*Ascaris* IgG and anti-*Ascaris* IgA in SI groups, at 0, 35, and 100 dpi. (C) Determination of the presence of anti-*Ascaris* IgG and anti-*Ascaris* IgA in RE groups at 0, 35, and 100 dpi. A one-way ANOVA test followed by Tukey’s test was used to evaluate differences among NI, SI, and RE groups in each dose (*n* = 8 per group). The results are shown as the mean ± SEM and statistical differences are represented by the asterisk, where * without the bar represents differences between the NI group and differences between the amount of exposure (SI × RE) in respective doses are represented by the asterisk with the bar and *p* < 0.05, **p < 0.01, and ***p < 0.001 or in B, the bar indicates differences between the groups in 35 and 100 dpi where (a) represents significant differences of the NI group, (b) 250 group, (c) 1250 group, and (d) 2500 group.

By 100 dpi, IgG levels declined in all SI groups, with only the SI 2500 group remaining significantly different from the control (Figure 8B), suggesting that a single exposure may not support long-term IgG production, especially at lower doses. In contrast, RE groups reached similar IgG levels at 35 dpi, but by 100 dpi, only RE 1250 exhibited a significant increase compared to 35 dpi, indicating a more sustained humoral response. RE 2500 maintained steady IgG levels over time (Figure 8C). Regarding suggesting that a single exposure may not support long-term IgG production IgA at 100 dpi, SI groups showed no differences across doses. However, in RE groups, those receiving 2500 eggs showed a significant increase in IgA levels compared to 35 dpi, with RE 2500 surpassing both control and lower dose RE groups (Figure 8C).

### 2.8 Principal Component Analysis Supports that the Lowest Doses after SI Do Not Immediately Enhance Immune Response and Result in Slower Repair and Persistent Damage

To further investigate the differences across experimental groups, principal component analysis (PCA) was performed using variables related to cellularity (leukogram, BAL leukocytes, NAG, EPO, MPO, and lung inflammatory infiltrates), host damage (hemoglobin, hematocrit, MCV, MCH, BAL hemoglobin, and total BAL proteins), and tissue repair (platelet count, fibrosis area, TGF-β, and IL-10 levels). The analysis revealed clear clustering patterns based on the infection dose and exposure frequency (Figure 9).

**Figure 9:**
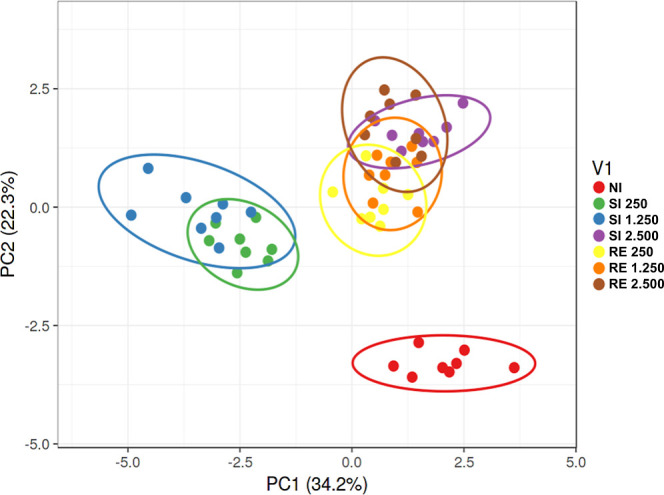
PCA of damage and repair mechanisms in BALB/c mice infected with *A. suum*. Analysis of observations of cellularity, host damage intensity, and repair mechanisms in different groups exposed to infection. Segregation between groups indicates distinct immune responses and repair patterns.

Notably, samples from the control group (NI) clustered distinctly from all infected groups, underscoring the specific host response triggered by the infection. A close association was observed between the SI 250 and SI 1250 groups, suggesting similar inflammatory and repair profiles under lower-dose, single-exposure conditions. In contrast, reinfected groups and the high-dose SI 2500 group clustered together, indicating comparable immune and pathological responses under conditions of repeated or intense exposure (Figure 9). This analysis not only highlights the variability between the groups but also provides information about the patterns of immune response and repair under different infection conditions.

## 3 Discussion

Experimental ascariasis has been studied with focuses on lung damage during peak larval migration,^2,10,19,20,25^ yet only two have assessed the effects postinfection,^21,26^ and only another two using different infectious doses,^23,24^ but still only during peak larval migration. Here, we demonstrated that larval ascariasis affects the host in different ways, and the prognosis, inflammatory response, and repair mechanisms are associated with the infectious dose and the number of exposures to the parasite. Furthermore, we also demonstrated that regardless of the dose, *Ascaris* infection can lead to the development of chronic pulmonary disease. This emerging evidence may contribute to future studies on larval ascariais as Amorim and colleagues^24^ have demonstrated, highlighting its potential relevance of the difference doses.

Initially, the mice’s weight gain and clinical signs revealed that those infected with the highest doses of eggs experienced faster weight loss and recovery with more pronounced clinical signs, regardless of the number of exposures. This is consistent with the results shown by Vieira-Santos and colleagues^27^ in SI 2500 mice. However, few studies evaluated weight variation at lower doses, except to observed by Lewis and colleagues,^23^ though they did not evaluate the mice over an extended period. We also observed delayed weight gain in mice infected with 250/1250 eggs, particularly in SI groups, which presented anemia at 35 dpi. Anemia could be detectable through blood counts, as reported by Cascio and colleagues,^28^ and is prevalent in children infected with geohelminths,^29–31^ including *Ascaris* spp.^32^ Recent data in the experimental model suggests that lower doses, like 250 eggs, more accurately mimic natural infection scenarios compared to higher doses like 2500 eggs.^24^

Our postpeak infection data raise important concerns about the long-term effects of ascariasis during childhood, a critical developmental stage predominantly affected by this parasite.^33^ In endemic areas, where coinfections such as malaria are common, anemia is frequently multifactorial and may worsen the clinical outcome. Indeed, a previous study demonstrated that concomitant *A. suum* and *Plasmodium berghei* NK65 infections exacerbated disease severity.^27^ In our model, mice infected with lower doses that more closely mimic the natural infection showed persistent anemia due to alveolar hemorrhage. Although alveolar hemorrhage has been previously reported in SI mice infected with *A. suum* as described by Nogueira and colleagues,^10^ anemia was not, likely due to earlier time points of analysis in these studies. Our findings suggest that such findings arise after the acute phase, highlighting the need for postpeak assessments.

Interestingly, in our study, anemia was not observed in the SI 2500 group at the time points analyzed. This does not exclude the possibility that mice infected with 2500 eggs developed anemia at some point during infection since at 8 dpi, these animals exhibited marked alveolar hemorrhage, and by 35 dpi, they showed improved hemoglobin levels compared to groups that received lower doses. In addition, these mice exhibited a significant increase in hemosiderin-laden macrophages in both lung tissue and BAL, similar to findings reported by Wu and colleagues^21^ at 100 dpi. This is supported by our histological observations and BAL analyses. Notably, we believe higher doses likely recruit more cells, aiding faster repair compared to lower doses. This suggests ongoing vascular damage and red blood cell extravasation, even in the absence of overt anemia. Persistent anemia likely contributes to fatigue, weakness, and delayed weight gain, which we observed in mice from lower-dose SI groups. Similar associations have been documented in children exposed to *Ascaris*.^29,34^ Overall, our data indicate that higher parasite loads and repeated exposures elicit stronger immune responses that accelerate repair mechanisms and weight recovery, as supported by improved BAL cellularity, reduced inflammatory infiltration, and normalization of body weight over time. In contrast, lower doses (250/1250 eggs) lead to persistent anemia and fibrosis, with delayed recovery and elevated platelet counts, highlighting a less efficient tissue repair process. These findings underscore the importance of considering both the dose and exposure frequency when evaluating the long-term impacts of larval ascariasis.

About the cytokine’s levels, mice infected with 2500 eggs exhibited cytokine levels largely comparable to the control group, except for IL-2, which was significantly decreased—most notably in the reinfected group. Notably, TGF-β levels were elevated in the reinfected group, while IL-6 levels were increased across all infected groups, regardless of the dose or number of exposures. IL-2 is a critical cytokine for T cell proliferation and differentiation into Th1, Th2, and Treg lineages, while also inhibiting Th17 polarization.^35^ We associate the observed IL-2 reduction—particularly in reinfected mice—with the elevated TGF-β levels, which are known to suppress IL-2 expression.^36^ Despite increased lymphocyte counts in the BAL and the presence of inflammatory infiltrates in this group, we suggest that these may reflect a residual response rather than ongoing active inflammation. At this time point, tissue repair mechanisms appear to rely predominantly on macrophages—evidenced by the abundance of hemosiderin-laden macrophages—and on eosinophils, potentially driven by IL-6 and elevated eosinophil peroxidase (EPO) activity. These findings suggest that high egg doses elicit a faster and more intense immune response than lower doses (250 and 1250 eggs), but this may come at the cost of tissue damage, leading to residual immune cell presence. In contrast, lower doses trigger slower, more prolonged immune activation. In the single-infection groups receiving 250 or 1250 eggs, we observed increased levels of Th2-related cytokines (IL-5 and IL-13) and the regulatory cytokine IL-10, all of which are central to helminth control.^37,38^ Previous studies have highlighted the roles of these cytokines in modulating helminth infections such as *Toxocara canis*,^39–41^ *Necator americanus*,^42,43^ *A. lumbricoides,* ^44^ and *A. suum*.^2,10^ IL-10, in particular, plays a vital immunoregulatory role not only in *A. suum* infection but also in *Trichuris muris* and *Schistosoma mansoni* infections by modulating Th1/Th17 responses.^45,46^ Moreover, IL-17 has been shown to contribute to helminth immunity in infections by *S. japonicum*,^47^ *T. canis*,^39,48^ and *A. suum*.^10^ However, the persistence of IL-17 in the lungs may also promote fibrosis—a link previously established in multiple studies.^10,47,49–51^

In our study, at 35 dpi, IL-5 and IL-13 levels decreased as egg dose increased. Interestingly, when mice were reinfected with the same dose, their cytokine levels aligned more closely with those of the control group. These findings suggest that a single exposure to low parasite doses fails to elicit a rapid and effective immune response. In contrast, at 100 dpi, IL-5 and IL-13 showed a dose-dependent increase, particularly in the reinfected groups, indicating a shift toward stronger type 2 responses with higher antigenic loads over time. Interestingly, while IL-10 remained relatively stable between doses at this later stage, TGF-β levels tended to decrease with an increasing egg dose, suggesting that regulatory constraints may loosen under intense or repeated antigen exposure, allowing Th2 effector activity to persist. This reversal in dose–response patterns between 35 and 100 dpi may reflect a transition from a partially downregulated immune environment during initial resolution (possibly to limit tissue damage) to an effector phase following chronic antigen persistence or reinfection.

The cytokine profile observed at 35 dpi supports the presence of a type 2 chronic pulmonary inflammatory condition that resembles an allergic airway disease. This aligns with the findings of Zhu and colleagues,^52^ who emphasized the role of IL-5 and IL-13 in such disorders. We observed significantly elevated levels of IL-5 and IL-13 in the SI 250/1250 groups at this time point. Although *Ascaris* larvae are no longer present in the lungs after 12 days,^2,21^ we hypothesize that persistent immune activation may be driven by parasite-derived proteins retained in the lung tissue, as proposed by Oliveira and colleagues.^26^ This hypothesis is supported by the persistent inflammatory infiltrates observed at 100 dpi and the elevated cytokine levels detected at that time. Although we did not employ the same methodology as Oliveira and colleagues^26^ to directly detect retained parasite antigens, our findings suggest that lower doses may favor antigen retention due to the slower and less intense immune response. This hypothesis should be validated in future studies. The capacity of *Ascaris* infections to induce type 2 responses and promote allergic lung pathology is well-established.^10,15,53,54^ Additionally, we observed an increase in IL-17 in the lungs of SI 1250 mice. IL-17 plays a key role in allergic airway disease pathogenesis by recruiting neutrophils and activating fibroblasts.^55,56^ Supporting this, we also noted increased neutrophil activity in this group, as evidenced by our BAL analysis.

The persistence of fibrotic areas led us to identify a pattern of altered respiratory parameters resembling obstructive pulmonary disease, as previously demonstrated by using a single-infection model with 2500 *A. suum* eggs.^21^ This was characterized by increased lung compliance and elasticity, evidenced by reduced lung resistance (Rl) and increased Cchord and Cdyn, as well as alterations in the Tiffeneau index (FEV50/FVC).^57,58^ The association between helminth infections and chronic lung diseases, such as emphysema, has been described in the context of *Nippostrongylus brasiliensis* infection.^59^ An important observation from our study is the presence of persistent lung damage at 100 dpi regardless of the infective dose. These findings suggest that varying burdens of *A. suum* eggs can induce a type 2 chronic pulmonary inflammatory pathology, marked by sustained fibrosis and long-term structural damage. While our histopathological analysis did not reveal classic emphysematous lesions, the pattern of lung injury (mainly parenchymal inflammation and fibrosis) points toward chronic respiratory dysfunction. Based on respiratory mechanics, this dysfunction resembles COPD; however, considering the histopathological profile and cytokine expression patterns, our model is more consistent with asthma-COPD overlap, a recently defined syndrome.^60^ This mixed pattern involves features of both obstructive and restrictive lung disease, as seen in *A. suum*-induced acute inflammation^10^ and pulmonary fibrosis^61^ with fibrosis particularly prominent at 100 dpi in the low-dose groups. Fibrosis is associated with increased collagen deposition in lung tissue,^62^ contributing to the observed increase in compliance, especially in the RE 250 group. We propose that delayed repair mechanisms at lower doses may lead to more extensive tissue damage over time, exacerbating the restrictive aspects of the disease by 100 dpi. It is important to highlight that while emphysema is the fourth leading cause of death worldwide^63,64^ and a known risk factor for other chronic conditions such as cancer,^65^ our findings do not support the development of emphysema per se in this model. Instead, they underscore *A. suum* infection as a driver of chronic pulmonary disease marked by persistent inflammation and fibrosis.

Considering our findings and those observed by Wu and colleagues,^21^ it is conceivable that, in addition to smoking, in endemic regions where *Ascaris* reinfections occur, ascariasis might be considered a risk factor for COPD with a pattern similar to emphysema. However, further studies, including those involving human subjects, are necessary to better understand this phenotype. Another important finding in our work was the similar levels of IgG at lower doses, also observed by Amorim and colleagues,^24^ and we demonstrate that these levels remain stable for up to 100 dpi. In addition to being favorable to the scenario of natural infection, this data may favor studies with low-dose vaccines minimizing host damage, comparing the damage caused after multiple exposures using the highest doses of eggs, as already demonstrated by us, by refs 10 and 24 . About IgA responses, evaluating this antibody can provide further information on mucosal immunity in long-term experimental ascariasi,specially in BAL, were it plays a crucial role in limiting parasite burden and preventing secondary infections by neutralizing antigens at mucosal surfaces.^66^ In our study, IgA levels were elevated across all infection scenarios when compared with noninfected controls, with the most pronounced increase observed in animals infected with 2500 eggs and subsequently reinfected. This pattern suggests that both infection intensity and repeated antigenic exposure enhance local mucosal immunity, potentially contributing to improved parasite control at mucosal sites.

This study, by assessing different infection doses, provided a broader view of the chronic immune response to *Ascaris* infection. However, some limitations need to be acknowledged. First, we used a restricted cytokine panel, which, although informative, may not capture the full complexity of the immune response; future studies could employ an expanded panel to explore the additional pathways involved. Second, while we selected specific collection points (8, 35, and 100 dpi) to represent acute, intermediate, and late infection stages, additional time points, such as 14 or 20 dpi to better capture the anemia phase and an intermediate point between 35 and 100 dpi (e.g., 70 dpi), could provide a more detailed picture of disease progression and recovery.

## 4 Conclusion

Our findings suggest that even low infectious doses, such as 250 eggs, can lead to persistent damage to the host, although less severe than higher doses. Lower doses seem to trigger a less intense immune response, potentially delaying clinical symptoms. Additionally, the humoral immune response to *A. suum* remains active for at least 100 dpi, possibly extending beyond this period. Notably, even with the low doses, two exposures triggered an immune response similar to that of the higher doses. These findings highlight the need for future research on the broader effects of ascariasis, especially its link to chronic inflammation and noncommunicable diseases and comorbidities, encouraging to develop new strategies for controlling ascariasis in endemic areas.

## 5 Materials and Methods

### 5.1 Ethics Statement

The maintenance of mice and all procedures performed during the experiments were conducted according to the Brazilian College of Animal Experimentation (COBEA) guidelines and approved by the local Ethics Committee for Animal Experimentation (CEUA) of the Universidade Federal de Minas Gerais (UFMG), Brazil, under protocol number 178/2021. All efforts were made to minimize animal suffering.

### 5.2 Mice

Specific pathogen-free female BALB/c mice (*Mus musculus*), aged 6 weeks old, were obtained from the Central Animal Facility of the UFMG, Brazil, and maintained at the Animal Facility of the Department of Parasitology of the UFMG under controlled conditions of temperature (24 ± 1 °C) and lighting (12 h light–dark cycle). During the experimental period, the mice had access to filtered water and commercial chow (Nuvilab Cr-1, Nuvital Nutrients, Brazil) *ad libitum*.

### 5.3 Parasites and Egg Culture

Adult *A. suum* worms were obtained from pigs at a Brazilian slaughterhouse (Belo Horizonte, Minas Gerais, Brazil). They were kept in PBS (0.4 M NaCl and 10 mM NaPO_4_) until processed at the Laboratory of Immunobiology and Control of Parasites at the UFMG, Brazil. The eggs were collected from the uterus of female worms, gently mechanically macerated, and then isolated using cell strainers (100 μm). The obtained eggs were incubated, according to Boes and colleagues,^67^ in 0.2 M H_2_SO_4_ for embryonation and maintained for 100 days in a BOD incubator at 26 °C, which corresponds to the peak of larval infectivity as described by Gazzineli-Guimarães and colleagues.^2^ After this period, larval eggs were used for experimental infection.

### 5.4 Experimental Design and Experimental Infection

For *A. suum* infection, on the day of inoculation, eggs were incubated with 5% (v/v) sodium hypochlorite solution and maintained at 37 °C in a humidified atmosphere of 5% CO_2_ for 2 h and washed with filtered water 3 times. Then, mice were infected via the intragastric route (gavage) with 0.2 mL of suspension containing a specific amount of larvated eggs for each group (250, 1250, or 2500) or only filtered water, followed by 0.1 mL of filtered H_2_O to wash the remaining eggs out of the syringe and needle as described by Gazzinelli-Guimarães and colleagues.^2^ Mice were randomized and divided into three groups: (1) the control or noninfection group (NI), which received filtered water by gavage; (2) the single-infection group (SI), which received only filtered water at day −14 and *A. suum* larvated eggs at day 0; and (3) reinfection group (RE), which were infected twice with larvated eggs of *A. suum* with an interval of 14 days (at days −14 and 0). In each group, mice were subdivided according to the different doses of larvated eggs of *A. suum* they received (250, 1250, and 2500) (Figure 1A). The utilization of a reinfection model using two exposures was based on results described by Amorim and colleagues.^24^ The mice were euthanized with an injection of xylazine/ketamine (8.5 and 130 mg/kg) on days 35 and 100 postinfection (dpi). All experiments were independently repeated twice.

### 5.5 Clinical Parameters and Score

To evaluate the clinical parameters, mice were followed up every 2 days, starting the day after the first infection up to day 80. The disease progression was evaluated by body weight changes and clinical scores, adapted from Vandermosten and colleagues.^68^ In this study, signals of piloerection (Pe), shivering (Sh), abnormal breathing (Ab), and trunk curling (Tc) were used to calculate the clinical score of the disease severity. A disease score of 0 (absent) or 1 (present) for Pe, Sh, Ab, and Tc was given, and a total clinical score (TCS) was calculated by the following formula: (TCS = Pe + Sh + Ab + Tc).

### 5.6 Parasitic Burden in Lung

Parasite burden was evaluated from infected mice by counting the total number of larvae recovered from the lungs and BAL as described by Lopes and colleagues.^69^ For this, at 8 dpi, the tissue was collected, sliced with scissors, and placed in a Baermann apparatus for 4 h in PBS at 37 °C and 5% CO_2_. In order to recover the larvae present in the airways, BAL was collected (see item Bronchoalveolar lavage BAL) and filtered in a 40 μm diameter cell strainer (BD Biosciences) to contain the larvae present in the BAL and collected in a 50 mL conical tube. The larvae recovered were fixed (10% formalin) and counted under an optical microscope at 10× magnification.

### 5.7 Hematological Analysis

Approximately 500 μL of blood was collected through the vena cava and transferred to tubes containing anticoagulant EDTA (Vacuplast, Brazil). An automated hematology counter (Bio-2900 Vet) was used for global leukocyte and platelet counts, hematocrit determination, hematimetric index, and hemoglobin dosage. For differential leukocyte counts, blood smears were made and stained with Giemsa, and 100 leukocytes were differentiated into lymphocytes, monocytes, eosinophils, and neutrophils under a light microscope at 100× magnification.

### 5.8 Bronchoalveolar Lavage

In order to perform the BAL, a 1.7 mm catheter was inserted into the trachea of mice, and 1 mL of cold sterile PBS was used to flush twice to BAL fluid. Samples collected were centrifuged at 300*g* for 10 min at 4 °C, and then the pellet was used to determine the total and differential counts (subpopulation of macrophages, lymphocytes, eosinophils, and neutrophils) under a light microscope at 100× magnification as described by Leal-Silva and colleagues.^40^ The supernatants were used for evaluation of alveolar hemorrhage by hemoglobin concentration (Bioclin, Quibasa, Brazil), total protein quantification using BCA Protein Assay Kit (Thermo Scientific, USA), as described by Nogueira and colleagues,^10^ and to quantify anti-*Ascaris* IgA levels (see Section 5.11 Extraction of Crude Antigen from *A. suum* L3 Larvae and Antibody-*Ascaris* Detection).

### 5.9 Tissue and Systemic Cytokine Profile

The right lung of each animal was used to determine the local cytokine profile, and it was homogenized using the tissue homogenizer PowerGen 125 (TissueLyser LT, Qiagen, Hilden, Germany) in extraction solution (0.4 M NaCl, 10 mM NaPO_4_, 0.05% Tween 20, 0.5% BSA, 0.1 mM phenylmethylsulfonyl fluoride, 0.1 mM benzethonium chloride, 10 mM EDTA, and 20 IU aprotinin A) at a rate of 1 mL per 100 mg of tissue. The homogenates were centrifuged at 8000*g* for 10 min at 4 °C; the supernatant was collected and stored at −80 °C until the cytokine quantification assay. The levels of IL-2, IL-4, IL-6, IL-10, IL-17A, IFN-γ, and TNF were assayed using cytometry bead array (Th1/Th2/Th17 BD Biosciences, USA) according to the manufacturer’s instructions. The data were acquired using an LSRFortessa flow cytometer (BD Biosciences, USA), and the results were analyzed in FlowJo software (Tree Star, Ashland, OR). IL-5, IL-13, and TGF-β were measured by the sandwich enzyme-linked immunosorbent (ELISA) kit (R&D Systems, USA) according to the manufacturer’s instructions. The absorbance of the samples was determined in a VersaMax ELISA microplate reader (Molecular Devices, USA) at a wavelength of 492 nm, and the cytokine concentration was calculated by interpolation using a standard curve fitted with five parameters of logistic (5-PL) and expressed in pg/mg of tissue for each sample.

### 5.10 Eosinophil Peroxidase, Neutrophil Myeloperoxidase, and n-Acetylglucosaminidase Assays

The activity of eosinophils, neutrophils, and macrophages was assessed by the concentration of EPO, MPO, and NAG, respectively, in pulmonary homogenate performed according to the method previously described by Leal-Silva and colleagues.^39^ Briefly, the tissues were homogenized according to Section 5.9 of this study. The homogenate was centrifuged at 8000*g* for 10 min at 4 °C; then, the pellet obtained was used to determine EPO, MPO, and NAG activity. The resulting pellet was processed according to refs 10 and 69 ; then, the absorbance was determined by a VersaMax ELISA Microplate Reader (Molecular Devices, USA) according to the previously published protocol for each assay, and the results are expressed as the optical density (O.D.).

### 5.11 Extraction of Crude Antigen from *A. suum* L3 Larvae and Antibody-*Ascaris* Detection

For the production of crude, antigen from L3 larvae was produced according to Lopes and colleagues.^70^ The supernatant resulting from the entire process was collected and stored at −80 °C until use. The amount of protein was measured using a commercial BCA kit (Thermo Fisher Scientific, USA) performed according to the manufacturer’s instructions. Anti-*Ascaris* total IgG antibodies were measured in plasma and *Ascaris*-specific IgA in BAL of *A. suum*-infected and noninfected mice. To measure antibodies, ELISA assays were performed in duplicate, as previously described by Gazzinelli-Guimarães and colleagues.^19^

### 5.12 Histopathological and Morphometric Analysis

The left lobe of the lungs was removed from the mice in each group to perform morphometric and semiquantitative analysis to evaluate inflammatory cells in tissue. The lungs were fixed in 10% formalin solution for 2 days, then gradually dehydrated in ethanol, diaphanized in xylol, and embedded in paraffin blocks that were cut at a thickness of 4 μm and fixed on microscopy slides. Slides with lung tissue were stained with hematoxylin and eosin (H&E) for the tissue damage and Masson’s trichrome stain to assess the fibrosis area. The tissue damage was described according to lesion intensity, inflammation, vascular phenomena, and cell profile.

For semiquantitative analysis, the slides were examined by two pathologists blinded to the experimental groups, and it was evaluated for airway inflammation score, vascular and parenchymal inflammation, and hemorrhage. The score was made according to the methodology previously described by Gazzinelli-Guimarães and colleagues,^19^ and the table score used is available in the Supporting Information (Table S1). Morphometric analysis was performed based on images captured from 20 randomly selected fields of the histological section per animal at 40× magnification, and the areas of inflammatory infiltrate were measured by counting the number of nuclei present in the histological sections using Image Pro Plus software, as described by Gazzinelli-Guimarães and colleagues.^19^ To evaluate the fibrosis area, 10 randomly selected fields were captured at 20× magnification and measured according to the total area stained by Masson’s trichrome as previously described.^71,72^ The images were obtained under a light field Olympus light microscope (BX-41) coupled from a capture system with a SPOT 3.4.5 Basic microcamera adapted.

### 5.13 Respiratory Function

Mice were anesthetized subcutaneously with ketamine and xylazine (09 and 130 mg/kg) to maintain spontaneous breathing under anesthesia, then tracheostomized and placed in a body plethysmograph, and connected to a computer-controlled ventilator (Forced Pulmonary Maneuver System, Buxco Research Systems, Wilmington, North Carolina, USA) as previously described by refs 10 and 73 . A respiratory rate of 160 breaths/min was set, and mechanical ventilation was conducted, which included the following: resistance and compliance tests were performed to determine parameters such as TV, dynamic compliance (Cdyn), and lung resistance (Rl). A quasi-static pressure–volume (PV) maneuver was performed to measure IC, followed by forced expiratory volume in 50 ms (FEV50), and forced vital capacity (FVC) was measured to calculate the Tiffeneau-Pinelli index (FEV50/FVC). Each parameter was obtained by performing three maneuvers to ensure reliable averages.

### 5.14 Statistical Analysis

GraphPad Prism 9.3.0 (GraphPad Software, Inc., USA) was used for statistical analysis. Grubb’s test was used to detect possible sample outliers. The Shapiro–Wilk normality test was performed to verify the distribution of the data. For comparisons between two groups, a *t*-test was applied, while evaluation between three or more groups was performed using ANOVA or Kruskal–Wallis test, followed by Tukey’s or Dunn’s post-test, respectively, according to the data distribution. A Pearson correlation test was used for the correlation analysis. For the PCA, different variables were analyzed using hierarchical clustering using the ClustVis 2.0 tool.^74^ All tests were considered significant at *p* ≤ 0.05.

## Supplementary Material



## Notes

Lung histopathological scoring system information; lung inflammation scores in lung tissue from *Ascaris suum*-infected mice at 35 and 100 dpi; and data from single and reinfected groups (PDF)
