# Supplementary material for: Larval Ascariasis Triggers Unresolved Anemia, Persistent Inflammation, and Chronic Pulmonary Disease after Single and Reinfection in a Dose-Dependent Manner in Mice
Source: ACS Infect Dis. 2025 Sep 12;11(10):2795–813. doi: 10.1021/acsinfecdis.5c00477 (PMC12519471; doi:10.1021/acsinfecdis.5c00477)
Supplement: Supplementary file 1 [file id5c00477_si_001.pdf]

Supporting information

Larval ascariasis triggers unresolved anemia, persistent inflammation, and chronic pulmonary disease after single and re-infection in a dose-dependent manner in mice

Jorge Lucas Nascimento Souza[a,d], Chiara Cássia Oliveira Amorim[a], Camila de Almeida Lopes[a], Flaviane Vieira-Santos[a], Ana Rafaela Antunes-Porto[a], Fernanda Rezende Souza[b], Evelyn Ane Oliveira[b], Thaynan Cunha Vieira[b], Lucas Kraemer[a], Marcelo Eduardo Cardozo[a], Ramayana Morais de Medeiros Brito[a], Luisa Mourão Dias Magalhães[c], Geovanni Dantas Cassali[b], Ricardo Toshio Fujiwara[a], Remo Castro Russo[d], Lilian Lacerda Bueno[a,*]

[a]Laboratory of Immunobiology and Control of Parasites, Department of Parasitology, Institute of Biological Sciences, Universidade Federal de Minas Gerais, Belo Horizonte, Brazil

[b]Laboratory of Comparative Pathology, Department of Pathology, Institute of Biological Sciences, Universidade Federal de Minas Gerais, Belo Horizonte, Brazil

[c]Laboratory of Interactions in Immunoparasitology, Department of Parasitology, Institute of Biological Sciences, Universidade Federal de Minas Gerais, Belo Horizonte, Brazil

[d]Laboratory of Pulmonary Immunology and Mechanics, Department of Physiology and Biophysics, Institute of Biological Sciences, Universidade Federal de Minas Gerais, Belo Horizonte, Brazil

***Author for correspondence:** Lilian Lacerda Bueno, E-mail: lilacerdabueno@gmail.com

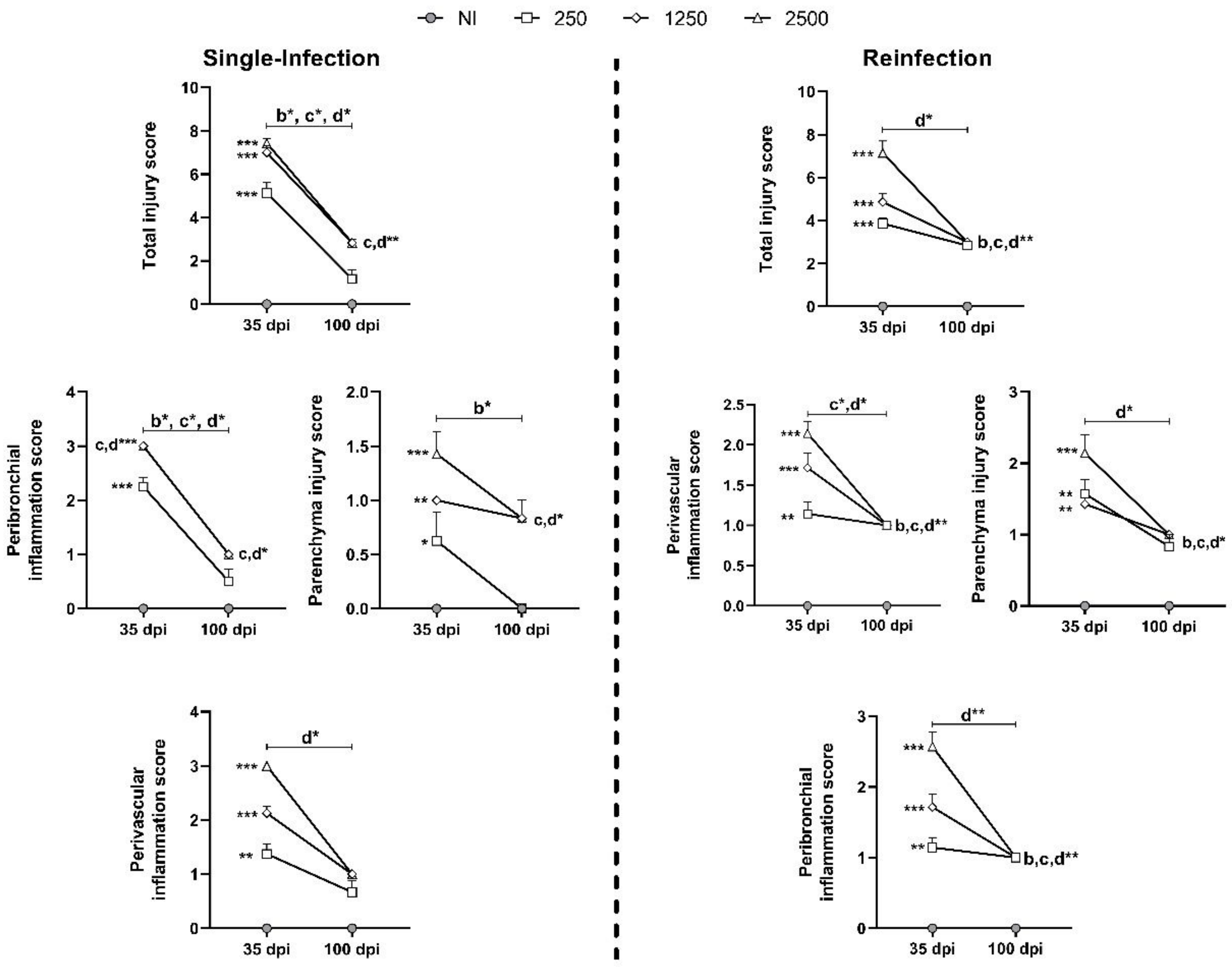

Figure S1: Scores of lung inflammation in lung tissue in mice infected with *A. suum* and evaluated at 35 and 100 dpi Evaluation of scores of lung inflammation in lung tissue of single-infected groups at 35 and 100 dpi and evaluation of scores of lung inflammation in lung tissue of reinfected groups at 35 and 100 dpi. Kruskal-Wallis test, followed by Dunn's test, was used to evaluate differences among NI, SI, and RE groups in each dose (n=8 per group) and between times. The results are shown as the mean ± SEM and statistical differences are represented by an asterisk, where * without the bar represents differences between the NI group at the same time or * with the bar indicates differences between the groups in 35 and 100 dpi where (a) represents significant differences of NI group, (b) 250 group, (c) 1,250 group, and (d) 2,500 group where $p < 0.05$, $^{**}p < 0.01$ and $^{***}p<0.001$.

Table S1: Lung histopathological scoring system

Semiquantitative histopathological lung analysis	
**Score**	**Airways inflammation score**
0	Absence of inflammatory cells around airways (absent)
1	Small number of cells around a few airways (mild)
2	A few airways have significant inflammation (moderate)
3	Most airways have some inflammation (pronounced)
4	Most airways are significantly inflamed (intense)
5	All airways are fully inflamed (severe)
**Score**	**Vascular inflammation score**
0	Absence of inflammatory cells around vessels (absent)
1	Small number of cells around a few vessels (mild)
2	A few vessels have significant inflammation (moderate)
3	Most vessels have some inflammation (pronounced)
4	Most vessels are significantly inflamed (intense)
5	All vessels are fully inflamed (severe)
**Score**	**Parenchymal inflammation**
0	≤ 1% of affected parenchyma
1	1-9% of affected parenchyma
2	10-29% of affected parenchyma
3	30-49% of affected parenchyma
4	50-69% of affected parenchyma
5	>70% of affected parenchyma
